# New gSSR and EST-SSR markers reveal high genetic diversity in the invasive plant *Ambrosia artemisiifolia* L. and can be transferred to other invasive *Ambrosia* species

**DOI:** 10.1371/journal.pone.0176197

**Published:** 2017-05-10

**Authors:** Lucie Meyer, Romain Causse, Fanny Pernin, Romain Scalone, Géraldine Bailly, Bruno Chauvel, Christophe Délye, Valérie Le Corre

**Affiliations:** 1 Agroécologie, AgroSup Dijon, INRA, Univ. Bourgogne Franche-Comté, Dijon, France; 2 Sustainable Agriculture Department, BASF France SAS, Ecully, France; 3 Department of Crop Production Ecology, Swedish University of Agricultural Sciences, Uppsala, Sweden; Consiglio Nazionale delle Ricerche, ITALY

## Abstract

*Ambrosia artemisiifolia* L., (common ragweed), is an annual invasive and highly troublesome plant species originating from North America that has become widespread across Europe. New sets of genomic and expressed sequence tag (EST) based simple sequence repeats (SSRs) markers were developed in this species using three approaches. After validation, 13 genomic SSRs and 13 EST-SSRs were retained and used to characterize the genetic diversity and population genetic structure of *Ambrosia artemisiifolia* populations from the native (North America) and invasive (Europe) ranges of the species. Analysing the mating system based on maternal families did not reveal any departure from complete allogamy and excess homozygosity was mostly due the presence of null alleles. High genetic diversity and patterns of genetic structure in Europe suggest two main introduction events followed by secondary colonization events. Cross-species transferability of the newly developed markers to other invasive species of the *Ambrosia* genus was assessed. Sixty-five percent and 75% of markers, respectively, were transferable from *A*. *artemisiifolia* to *Ambrosia psilostachya* and *Ambrosia tenuifolia*. 40% were transferable to *Ambrosia trifida*, this latter species being seemingly more phylogenetically distantly related to *A*. *artemisiifolia* than the former two.

## Introduction

Inferring recent demographic history and contemporary evolutionary processes are major goals in the field of population genetics. Climate change, human disturbances of natural habitats and human-aided dispersal can cause dramatic shifts in the distributions of natural species, and biological invasions are increasingly prevalent worldwide. Analyzing the genetic diversity and population genetic structure of native and introduced populations of an invasive species allows recovering pathways of invasion and identifying founding events and/or admixture events among invasive populations. All these processes affect the demographic success and future expansion of the invasive species and determine its potential for adaption to new environmental conditions. Their understanding is invaluable for devising appropriate management strategies [1,2].

The molecular markers used for population genetics studies are currently essentially of two kinds: genome-wide Single Nucleotide Polymorphisms (SNPs) identified by next-generation-sequencing-based techniques such as Restriction site Associated DNA sequencing (RAD-seq) or genotyping-by-sequencing [3], and Simple Sequence Repeats (SSR) markers (microsatellites). Some limitations of SSR markers are low density throughout the genome, complex mutational patterns and possible presence of homoplasy and null alleles [4]. However, SSR markers are easy to score, highly polymorphic and thus highly informative and the theory and practice of SSR marker analysis and their afferent bias are well known [5], making them still the markers of choice for ecological and evolutionary studies. In comparison to SNPs, SSRs are especially well suited for analyzing processes occurring at small temporal or spatial scales and have proven highly relevant for revealing recent expansion and recent admixture or analyzing parentage and kinship [5–7]. Next-generation sequencing technologies now allow to rapidly develop large sets of SSRs [8]. In addition, the ever-increasing availability of transcriptome sequences (Expressed Sequence Tags, EST) in public databases enables fast and cost-effective development of genic SSRs marker (EST-SSRs). EST-SSRs are expected to be less polymorphic than gSSRs but also to display fewer null alleles and be more transferable among related species [9,10]. As their polymorphism may be influenced by selective processes, EST-SSRs may reveal somewhat different genetic patterns than gSSRs [11].

The genus *Ambrosia* in the *Asteraceae* family includes at least 51 species collectively known as “ragweeds” and mainly distributed in North America [12]. Four different species (*A*. *artemisiifolia* L., *A*. *trifida* L., *A*. *psilostachya* D.C. and *A*. *tenuifolia* Spreng.) occur in Europe but are native from America [13,14]. *A*. *artemisiifolia* is an annual herb mostly known as a successful invasive and a highly allergenic plant causing severe rhinitis and asthma [15,16]. It has been introduced in Europe in the 19^th^ century by the import of contaminated grain and forage [17]. *A*. *artemisiifolia* has colonized different types of habitats such as railways, riversides and wastelands, as well as cultivated fields where it is now a noxious weed competing with several summer crops [17]. To investigate the population genetic structure in *A*. *artemisiifolia*, reliable and polymorphic molecular markers are needed. To date, only a few gSSR markers have been developed from French *A*. *artemisiifolia* populations [18,19]. These few gSSRs were used to assess the population genetic structure and patterns of colonization across continental and regional scales in Europe [20–25], North America [26] and China [27]. In addition, most of the gSSR markers available showed PCR amplification failures and excess homozygote genotypes [20–26]. Excess homozygosity can be caused by the presence of null alleles resulting from mutations at primer binding sites that preclude PCR amplification. Alternatively, excess homozygosity has sometimes been interpreted as evidence for partial selfing in a mostly outcrossing species. This issue was highly debated in several SSR-based population genetics studies conducted on *A*. *artemisiifolia* [20–23].

The present study had three purposes: (a) develop new nuclear SSR markers for *A*. *artemisiifolia* following three different approaches (whole-genome enrichment followed by 454 sequencing, whole-genome Illumina sequencing, and use of existing EST databases), (b) investigate the genetic diversity, population structure and mating system of *A*. *artemisiifolia* using populations sampled in North America and Europe, and (c) assess marker transferability to *A*. *trifida*, *A*. *psilostachya* and *A*. *tenuifolia*.

## Materials and methods

### Plant material

**Fig 1:**
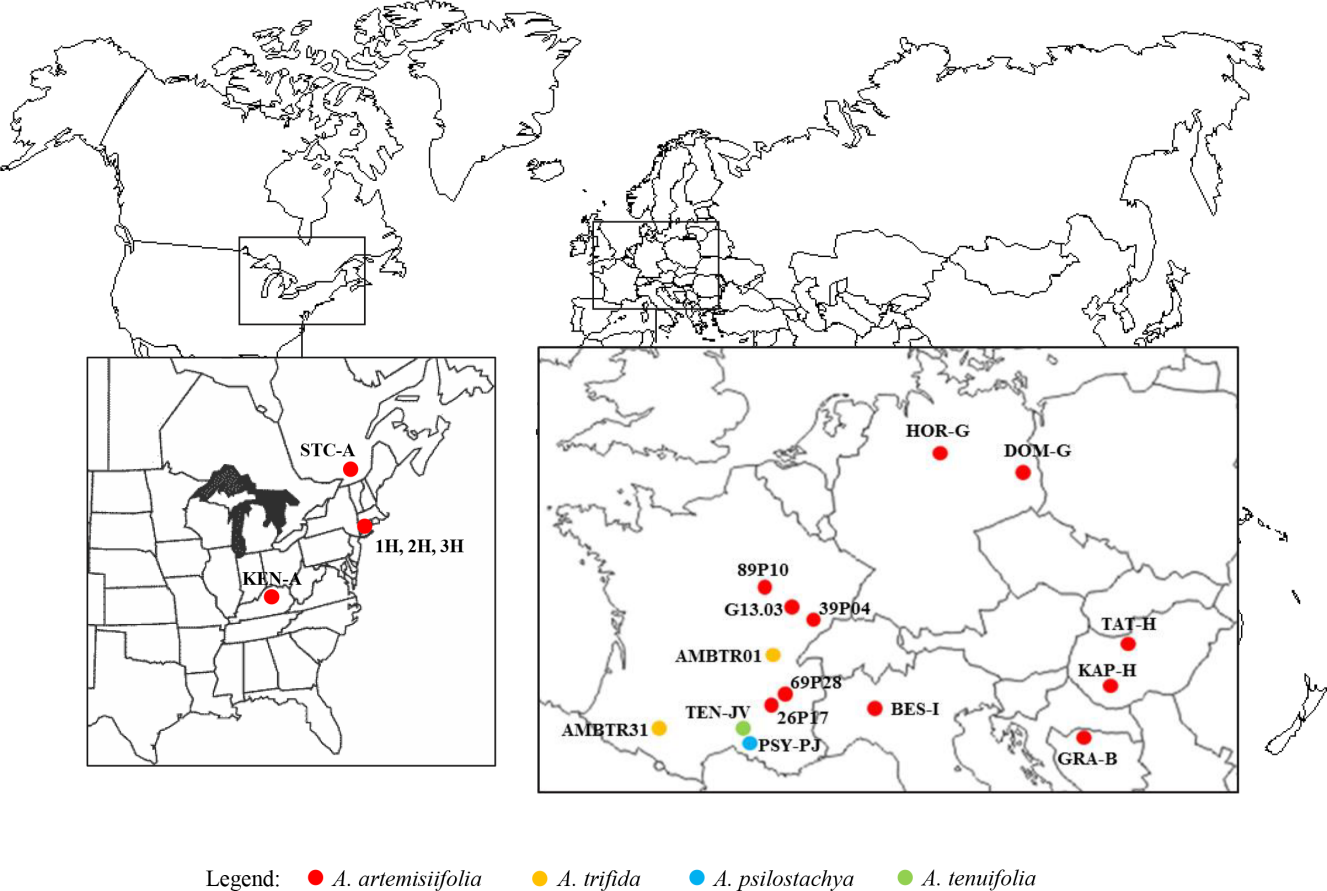
Map of the studied *Ambrosia sp*. populations.

A total of 321 *A*. *artemisiifolia* individuals were sampled from 11 populations spanning the invasive range in Europe and 5 populations in North America (Table 1, Fig 1). Twenty individuals were sampled from two populations of *A*. *trifida*, 22 individuals from one population of *A*. *psilostachya* and 21 individuals from one population of *A*. *tenuifolia*. A 0.2-cm^2^ leaf section was collected on each individual and DNA extracted as described in [28]. All three species studied are alien invasive, not protected species. Sampling locations were not localized within protected areas so that no specific permission was required. *Ambrosia artemisiifolia* is described as a diploid species (2n = 36, [13, 29,30]). As the presence of triploid plants has sometimes been questioned [26], we counted nuclear chromosomes as described [31] in 10 plants randomly chosen from one French population. Results were in agreement with diploidy with 2n = 36. (S1 Fig). *Ambrosia trifida* is a diploid species with a different basic chromosome number (2n = 24) [29, 30], while *A*. *psilostachya* and *A*. *tenuifolia* have the same basic chromosome number as *A*. *artemisiifolia* but variable ploidy levels [13,14, 29, 32].

**Table 1:** *Ambrosia sp*. populations analyzed.

Species	Population code	Country	Date of sampling	Nb of individuals analyzed	Geographic coordinates
*Ambrosia artemisiifolia*	1H	USA	2013	16	Not available^a^
	2H	USA	2013	11	Not available^a^
	3H	USA	2013	15	Not available^a^
	KEN-A	USA	2010	24	N38°01’00”, W84°33’10”
	STC-A	Canada	2010	20	N45°10’03”, W73°40’50”
	26P17	France	2005	24	N44°44’52”, E04°55’07”
	39P04	France	2011	24	N46°45’56”; E05°34’10”
	69P28	France	2013	24	N45°44’49”, E05°04’59”
	89P10	France	2011	24	N48°10’40”, E03°15’02”
	GEN13.03	France	2013	24	N47° 11' 29”, E05° 15' 0”
	BES-I	Italy	2011	20	N45°18’25”, E08°58’21”
	HOR-G	Germany	2011	11	N52°17’28”, E10°38’26”
	DOM-G	Germany	2011	24	N51°38’21”, E14°11’50”
	TAT-H	Hungary	2009	20	N47°34’21”, E18°27’18”
	KAP-H	Hungary	2011	20	N46°22’12”, E17°51’17”
	GRA-B	Bosnia	2011	20	N45°08’19”, E17°15’51”
*Ambrosia trifida*	AMBTR01	France	2013	3	N46°24’35”, E5°5’42”
	AMBTR31	France	2013	17	N43°15’48”, E1°2’36”
*Ambrosia psilostachya*	PSI-PJ	France	2014	22	N44°25’42” ; E04°42’44’
*Ambrosia tenuifolia*	TEN-JV	France	2014	21	N43°49’54” ; E04°34’10’

^a^These populations were sampled in Connecticut, USA.

### Development of new nuclear SSR markers for *A*. *artemisiifolia*

#### Obtaining sequence data

For the SSR-enriched gDNA library approach, total gDNA from 8 *A*. *artemisiifolia* individuals was isolated using the DNeasy Plant Mini Kit (QIAGEN, Valencia, California, USA) and processed by GenoScreen, (Lille, France). A SSR enriched DNA library was obtained as described in [33]. Briefly, total DNA was mechanically fragmented and enriched for AG, AC, AAC, AAG, AGG, ACG, ACAT and ATCT repeat motifs. Enriched fragments were subsequently amplified. Amplicons were sequenced on a 454 GS FLX Titanium system (454 Life Sciences, Branford, USA) following manufacturer’s protocols.

For the Illumina gDNA sequencing approach, total gDNA from one *A*. *artemisiifolia* individual was extracted using the DNeasy Plant Mini Kit (QIAGEN, Valencia, California, USA). Sequencing was performed at GENTYANE (INRA, Clermont-Ferrand, France). An Illumina paired-end shotgun library was prepared by shearing 2 μg DNA using a Covaris M220 ultrasonicator (Covaris, Woburn, USA) and following the standard Illumina TruSeq DNA Library Kit protocol (Illumina, San Diego, USA). Sequencing was conducted on the Illumina MiSeq with 250 bp paired-end reads.

For the EST public data use approach, the two existing sets of *A*. *artemisiifolia* transcriptome 454 sequence data were downloaded from Genbank Sequence Read Archive [34]. They correspond to one individual sampled in the USA (accession SRX096892) and one sampled in Hungary (accession SRX098769). Both datasets were merged before analysis.

For each of the three sequence datasets, stringent sequence quality control and filtering were performed using the ShortRead package in the Bioconductor software [35]. Briefly, read ends were first trimmed by quality scores. Only sequences longer than 300 bp (454 reads) or 200 bp (Illumina reads) with a mean Phred quality score higher than 30% and less than 1% Ns were retained. Exact sequence duplicates were discarded. In the Illumina dataset, only matching paired-end reads were kept after quality filtering and overlapping reads were merged using FLASH [36]. Detection of SSR motifs was conducted on the merged reads only, ensuring that the size of the flanking regions was large enough to design good-quality primers.

#### SSR identification and primer design

SSRs were identified with QDD version 3.1 [37]. Only 2- to 6-nucleotides motifs were considered. The minimum repeat unit was set to eight for di-nucleotides, six for tri-nucleotides, and five for longer motifs. Expected amplicon sizes were constrained to a 100–300 bp range. Primer pairs were thoroughly tested for clear, stable amplification on 12 *A*. *artemisiifolia* individuals from three populations (one French population from the Rhône Valley, the German population DOM and the American population KEN). PCRs were performed in 10-μL as previously described [28]. Cycling parameters consisted in a first denaturation step (2 min at 95°C) followed by 39 cycles of 5 s at 95°C, 10 s at 60°C and 30 s at 72°C. Amplicons were visualised by electrophoresing five microliters of PCRs on 3% (wt/vol) agarose gels run for 25 min at 100V in Tris-Borate EDTA buffer.

### SSR marker validation and assessment of genetic polymorphism in *A*. *artemisiifolia*

#### Genotyping

SSRs successfully amplifying in *A*. *artemisiifolia* were used to genotype 384 individuals, including 321 *A*. *artemisiifolia* (16 populations), 20 *A*. *trifida* (two populations), 22 *A*. *psilostachya* (one population) and 21 *A*. *tenuifolia* (one population) individuals (Table 1). Genotyping was performed at GENTYANE (INRA, Clermont-Ferrand, France). PCR products were labelled with one fluorescent tag (6-FAM, NED, VIC or PET) and loaded on an ABI 3730XL capillary DNA analyzer (Applied Biosystem) with the size standard GS500 LIZ. Peakscanner version 1.0 (Applied Biosystems) and the R package MsatAllele were used to read allele sizes [38]. A Principal Component Analysis (PCA) was performed on genotype data using the package adegenet [39] in R 3.1.2 in order to examine the genetic relationship among the four species studied.

#### Check for null alleles

MicroChecker 2.2.0.3 was used to check for the presence of null alleles and scoring errors due to stuttering and large allele dropout for each marker in each *A*. *artemisiifolia* population [40]. The markers showing the overall lowest occurrence of null alleles and stuttering were retained for further analyses. Frequencies of null alleles at the retained loci in each population were estimated using INEST 2.1 [41].

#### F_ST_ outlier tests

All SSR loci were screened for evidence of selection based on an F_ST_ outlier test that identifies loci with an F_ST_ value unexpectedly high (diversifying selection) or unexpectedly low (balancing or purifying selection). We used data from *A*. *artemisiifolia* and the software Bayescan [42]. This program implements a Bayesian method based on a multinomial Dirichlet distribution for allele frequencies. The Dirichlet distribution holds under a variety of demographic models when populations derive from a common gene pool. As a recent range expansion has been shown to increase the proportion of false selection event detection [43], we used a conservative prior value of 100 for the ‘odds of neutrality’ (only 1 locus out of 100 was under selection). For each locus, probability for selection was examined based on relative posterior probabilities for models with and without selection. We implemented 20 pilot runs of 5,000 iterations, a burn-in period of 50,000 iterations and 100,000 subsequent iterations with a sample size of 5,000 and thinning interval of 20.

### Estimation of the mating system in *A*. *artemisiifolia*

The mating system of *A*. *artemisiifolia* was investigated using five gSSR markers (SSR10, SSR17, SSR47, SSR71 and SSR73) in six additional French populations sampled in 2014 and located within a few kilometres around population GEN13.03 (Table 1). These gSSR markers showed less null alleles than others. Leaf tissue and mature seeds were collected on six to eight mother-plants per population. Eight to 16 progeny-plants per mother-plant were genotyped, yielding a total of 614 individuals. MLTR [44] was used to estimate the multi-locus outcrossing rate *tm*, the maternal inbreeding coefficient *F*, the outcrossing rates between related individuals *tm-ts* and the correlation of paternity *rp*.

### Genetic diversity and inbreeding

The allelic richness per locus and per population using a rarefaction method (*A*), expected heterozygosity (*H*_S_) and the genetic differentiation (*F*_ST_) were calculated using Fstat [45]. Significance of *F*_ST_ values was based on 1000 bootstrap resampling over loci. Inbreeding coefficient (*F*_IS_) were estimated with INEST 2.1 [41] using a Bayesian procedure robust to the presence of null alleles. To assess the statistical significance of inbreeding we compared the model with inbreeding with the random mating model (*F*_IS_ = 0) based on the Deviance Information Criterion (DIC). Genetic diversity and differentiation parameters for *A*. *artemisiifolia* were calculated over all populations, over North American populations and over European populations.

### *A*. *artemisiifolia* population structure

Population structure was assessed using Structure 2.2 [46]. The admixture model and correlated allele frequencies between populations were selected as specified [47] to determine the number of genetic clusters (*K*) best fitting the data. The length of the burn-in period was 100,000 runs followed by 500,000 Markov Chain Monte Carlo. Ten iterations were performed for each value of *K* from 1 to 15. *K* was determined graphically based on log likelihood values as previously described [46] using the web-based program Structure Harvester [48]. In addition, the ΔK method [49] was used to determine the best value of *K*. Finally, Clumpp 1.1.2 [50] and R 3.1.2 were used to produce graphical outputs for the inferred population structure.

### Genetic divergence and bottleneck tests

Genetic divergence is likely to vary across populations because of differences in population effective sizes and local migration rates. This is especially the case when recent founder effects have occurred, such as during range expansions. Patterns of genetic divergence were estimated for the invasive range (Europe) by calculating population-specific *F*_ST_ values based on the *F*-model [51]. We used the Bayesian method of Foll and Gagiotti [52] implemented in the software GESTE v2. To assess geographical patterns in genetic divergence, we compared three models: one null model that simply estimate population-specific *F*_ST_ values, and two models that used either latitude or longitude as explanatory variables. In addition to the study of genetic divergence patterns, we investigated the signature of recent bottleneck events using the Wilcoxon test for excess expected heterozygosity implemented in INEST2.1 and based on the method of Cornuet and Luikart [53]. Analyses were run with the Two-Phase Mutation (TPM) model with default settings.

## Results

### Development of new nuclear SSR markers

Sequencing results, filtering and success rates of microsatellite loci development are summarized in Table 2. Most 454 reads were eliminated because of insufficient length, while most Illumina reads were eliminated because paired-end reads could not be merged. As expected, the proportion of quality reads containing a SSR motif was much higher among the 454 reads obtained from an enriched gDNA library (24%) than among the Illumina reads obtained from raw gDNA (0.3%) or among the transcriptome 454 reads (0.1%). The low rate of SSR motifs obtained from Illumina sequencing of raw gDNA was more than compensated for by the high amount of reads generated and this method allowed the identification of ten times more potentially amplifiable loci than 454 sequencing of enriched gDNA (Table 2). The distribution of motif length was very similar between the two methods used to develop gSSRs: on average the di- tri-, tetra-, penta- and hexa-nucleotides accounted for 40.2%, 48.7%, 7.5%, 2.5% and 1% of gSSRs, respectively. By contrast, most EST-SSRs were tri-nucleotides (81.6%), and di-, tetra-, penta- and hexa-nucleotides accounted for 11.1%, 7%, 0.2% and 0% of EST-SSRs, respectively. Success rate of PCR amplification were quite similar among SSR sets, yielding 67 gSSRs (GenBank accession number KX867678—KX867743) and 41 EST-SSRs (GenBank accession number KX867744-KX867785) with consistent amplification in *A*. *artemisiifolia* (Table 2). Among these, 46 gSSRs and 32 EST-SSRs gave clear, easy to score patterns after capillary electrophoresis (S1–S3 Tables). Homology with known proteins was detected for 25 EST-SSRs (S3 Table).

**Table 2:** Characteristics of sequence datasets used for development of new SSR markers.

Sequence dataset	Total number of reads	Number of good quality reads (%)	PALs^a^	Number of tested loci	Number of loci successfully amplified (%)
Enriched Genomic—454	556,018	18,218 (3.27%)	270	96	30 (31.2%)
Genomic—Illumina	12,000,000	923,229 (7.69%)	2,720	110	37 (33.6%)
EST—454	1,317,778	393,302 (29.85%)	397	173	41 (23.7%)

^a^ Potentially Amplifiable Loci (PALs).

### Genetic polymorphism at gSSRs and EST-SSRs loci in *A*. *artemisiifolia*

Genetic polymorphism was assessed using 16 *A*. *artemisiifolia* populations for a total of 321 individuals (Table 1). After checking for null alleles and stutters at each locus in each population, 14 gSSRs and 13 EST-SSRs were retained as best markers for population genetic analysis. Among the 27 loci tested, only one (SSR86, S1 Table) was unambiguously detected as being under selection (Bayesian probability = 1, S2 Fig). SSR86 showed less genetic differentiation (F_ST_ = 0.014) than other markers, but a very high within-population genetic diversity (Hs = 0.776), suggesting balancing selection at or near the locus. This locus was therefore discarded for further analyses.

**Fig 2:**
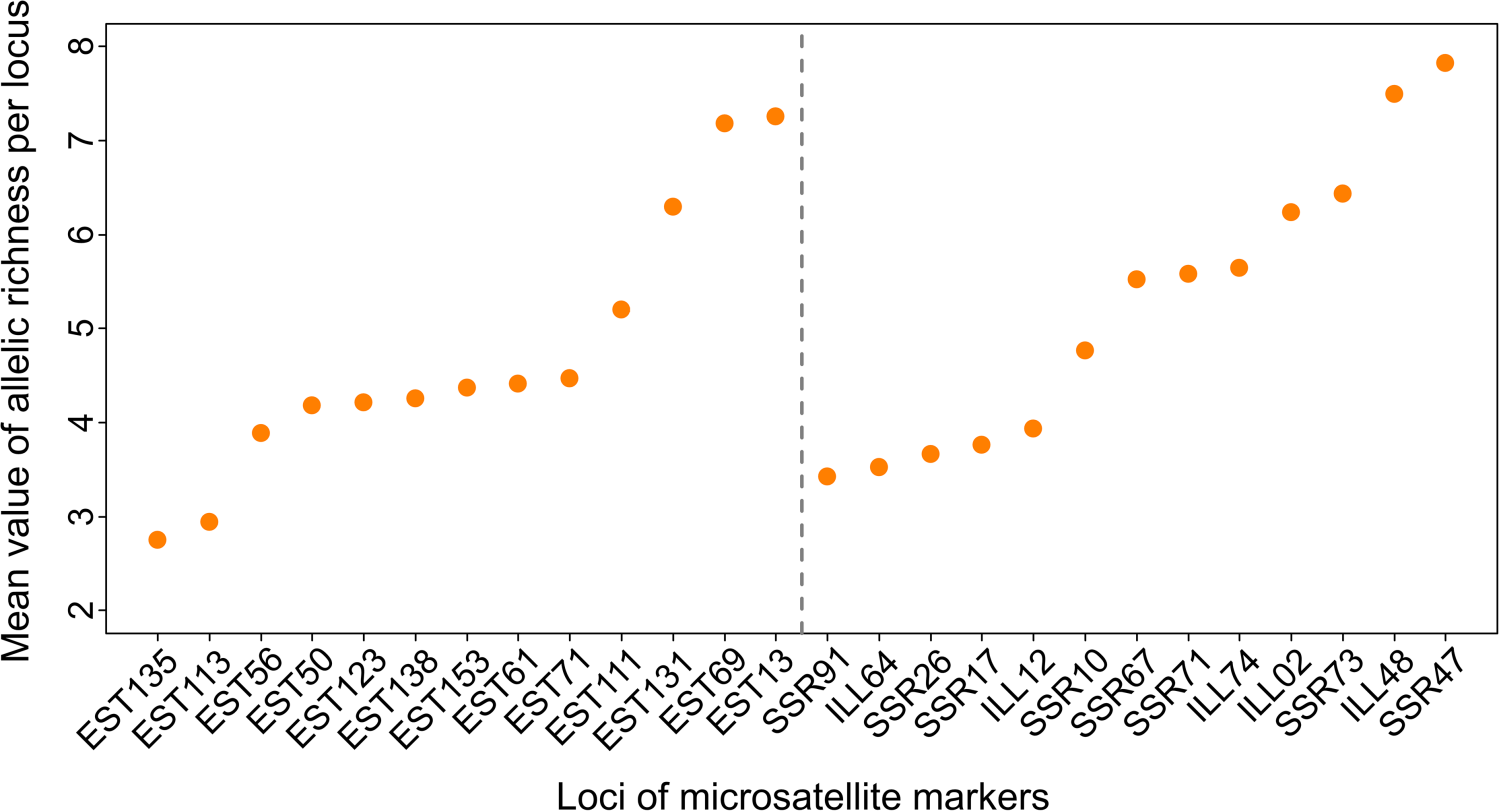
Allelic richness for the 13 EST-SSRs and 13 gSSRs analyzed in *A*. *artemisiifolia*. Markers are plotted by increasing mean value.

All 26 retained loci were polymorphic and revealed high levels of genetic diversity (Table 3). The frequencies of null alleles estimated over all populations ranged between 0.06 and 0.19 and were on average similar between gSSRs and EST-SSRs (0.11; Table 3). Allelic richness per locus and population, calculated based on a minimum sample size of eight individuals (Fig 2), was slightly lower for EST-SSRs than for gSSRs (4.438 *versus* 4.748) but the difference was not significant (Wilcoxon test p-value = 0.778) (Fig 2). The mean expected heterozygosity within populations was high (0.635 for gSSRs and 0.625 for EST-SSRs) and not significantly different between the two types of markers (Wilcoxon test p-value = 0.778). The mean genetic differentiation F_ST_ was 0.072 for gSSRs and 0.058 for EST-SSRs (difference significant, Wilcoxon test p-value = 0.045).

**Table 3:** Genetic diversity and differentiation estimated at 13 gSSR and 13 EST-SSR loci in 16 populations of *A*. *artemisiifolia*. Number of alleles (*N*a), mean observed (*H*o) and expected (*H*s) heterozygosity, genetic differentiation (*F*_ST_) and average across-populations frequency of null alleles (*P*null) are indicated for each marker.

Locus	Repeated motif	Size range	Na	Ho	Hs	*F*_ST_	*P*null
**ILL02**	(ACCACT)_6_	265–297	12	0.495	0.605	0.054	0.108
**ILL12**	(AACAG)_5_	114–148	10	0.309	0.432	0.095	0.163
**ILL48**	(AGC)_10_	116–148	14	0.523	0.702	0.053	0.087
**ILL64**	(ACC)_7_	279–304	8	0.501	0.538	0.104	0.073
**ILL74**	(AGC)_7_	281–308	11	0.662	0.740	0.067	0.081
**SSR10**	(GATA)_6_	160–203	10	0.378	0.625	0.071	0.194
**SSR17**	(CATA)_5_	145–196	9	0.320	0.485	0.105	0.135
**SSR26**	(GAA)_9_	106–120	6	0.566	0.588	0.074	0.072
**SSR47**	(AG)_9_	96–136	20	0.488	0.762	0.066	0.127
**SSR67**	(GAA)_7_	183–230	14	0.580	0.707	0.091	0.105
**SSR71**	(TCC)_7_	127–161	12	0.715	0.706	0.065	0.071
**SSR73**	(AC)_8_	185–206	13	0.640	0.772	0.044	0.091
**SSR91**	(TC)_8_	108–118	6	0.537	0.592	0.072	0.159
**EST13**	(AGT)_9_	173–225	18	0.594	0.814	0.050	0.089
**EST50**	(AAAG)_5_	204–224	8	0.451	0.634	0.052	0.160
**EST56**	(AGC)_7_	208–223	5	0.647	0.688	0.052	0.070
**EST61**	(AAT)_7_	144–180	13	0.471	0.653	0.048	0.105
**EST69**	(AAT)_7_	116–138	12	0.614	0.761	0.070	0.090
**EST71**	(AAT)_7_	141–165	13	0.394	0.545	0.054	0.098
**EST111**	(ACC)_7_	134–159	9	0.491	0.625	0.099	0.087
**EST113**	(ACC)_7_	151–167	6	0.427	0.516	0.056	0.137
**EST123**	(ACC)_7_	114–142	8	0.374	0.589	0.064	0.165
**EST131**	(ACC)_7_	117–131	13	0.456	0.705	0.051	0.114
**EST135**	(ACG)_7_	132–148	6	0.419	0.456	0.043	0.073
**EST138**	(ACC)_7_	130–168	12	0.283	0.498	0.065	0.140
**EST153**	(AGG)_6_	172–193	8	0.641	0.645	0.052	0.058

### Insight into the mating system in *A*. *artemisiifolia*

Thirty-six maternal progenies sampled from six French populations were analysed with five gSSRs to estimate mating system parameters. In addition, direct evidence for the presence of null alleles was sought by considering the progenies from maternal plants apparently homozygous at one locus. If a null allele was present, the maternal plant would actually be heterozygous (i.e., carrying one null allele and one detectable allele). Its progeny would thus contain some plants apparently homozygous for alleles different from the maternal one, but actually carrying one maternal null allele and one paternal detectable allele. Evidence for the presence of null alleles was obtained for all five markers. Depending on the marker considered, from 25% (3 out of 12) to 35% (8 out of 23) of the progenies from plants scored as homozygotes contained non-matching genotypes. Mating system parameters were estimated after excluding these progenies. The maternal inbreeding coefficient was non-significant (F = 0). Multi-locus outcrossing rates (tm) were high and not significantly lower than 1 in all populations (Table 4). The rates of mating between related individuals (tm-ts) were not significant. Paternity correlations (rp) were weak and only significant for two populations. These results suggested complete outcrossing for *A*. *artemisiifolia* and large numbers of pollen donor parents.

**Table 4:** Estimates of mating system parameters for six French *A*. *artemisiifolia* populations based on five SSR markers (SSR10, SSR17, SSR47, SSR71 and SSR73). Multi-locus outcrossing (tm) and single-locus outcrossing (ts) rates, outcrossing rate between related individuals (tm-ts), maternal inbreeding coefficient (F) and correlation of paternity (rp) are indicated for each marker.

Samples	*tm*	*ts*	*tm—ts*	*F*	*rp*
GEN02	1.025 (0.080)	1.065 (0.083)	-0.040 (0.059)	-0.200 (0.058)	0.151* (0.049)
GEN05	0.882 (0.114)	0.789 (0.115)	0.093 (0.037)	-0.037 (0.104)	0.001 (0.179)
GEN07	0.941 (0.077)	0.895 (0.077)	0.046 (0.046)	-0.072 (0.152)	0.106 (0.061)
GEN10	0.963 (0.056)	0.906 (0.085)	0.057 (0.046)	-0.200 (0.046)	0.063 (0.048)
GEN11	0.985 (0.081)	0.933 (0.133)	0.052 (0.073)	-0.125 (0.130)	0.175* (0.086)
GEN17	0.762 (0.161)	0.693 (0.118)	0.069 (0.062)	-0.110 (0.047)	0.118 (0.074)

The values in brackets are S.D.

* indicates significant values.

### Patterns of genetic diversity and inbreeding in *A*. *artemisiifolia* populations

We compared patterns of genetic diversity between the native range (North America) and the invasive range (Europe). Allelic richness within population and mean expected heterozygosity within population were slightly higher in North America than in Europe (Table 5). However, for both parameters, the difference between the two ranges was not significant (Wilcoxon test p-values: 0.100 and 0.173 for allelic richness and expected heterozygosity, respectively). The inbreeding coefficient estimated taking null alleles into account was significantly higher than zero in only seven populations: four of the five North-American populations and three of the eleven European populations (S4 Table). Consequently, *F*_IS_ values were on average higher in the native range than in the invasive range (Table 5), although the difference was not significant (Wilcoxon test p-values: 0.2951). F_ST_ in the native range was low (0.042) but significant. F_ST_ in the invasive range was higher (0.071) and also significant. The difference in F_ST_ values between the two areas was significant based on 99% bootstrap confidence intervals.

**Table 5:** Genetic diversity parameters across populations of *A*. *artemisiifolia* sampled within (i) North America and Europe, (ii) North America only and (iii) Europe only. *A*: average allelic richness after rarefaction, *H*_O_: observed heterozygosity, *H*_S_: expected heterozygosity, *F*_IS_: inbreeding coefficient estimated taking into account the presence of null alleles, *F*_ST_: coefficient of genetic differentiation among populations.

Group	Number of populations	*A*	*H*_O_	*H*_S_	*F*_IS_	*F*_ST_
Overall	16	3.989	0.544	0.630	0.078	0.064*
North America	5	4.193	0.651	0.651	0.092	0.042*
Europe	11	3.896	0.496	0.620	0.065	0.071*

* *F*_ST_ estimate significantly higher than zero based on 99% bootstrap confidence intervals.

### Population genetic structure of *A*. *artemisiifolia*

**Fig 3:**
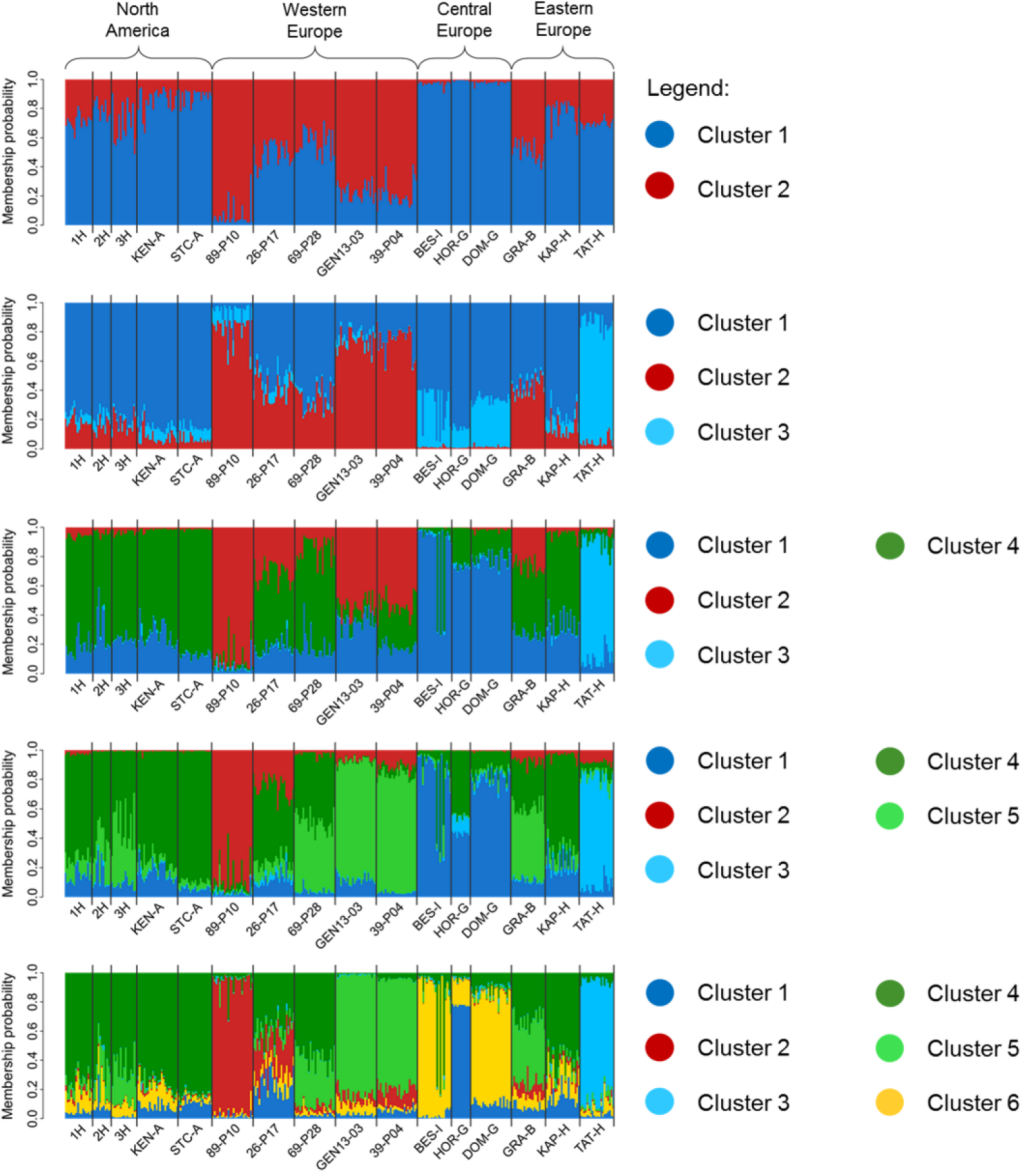
Individual plant membership probabilities for the genetic clusters identified by the software Structure within 16 *A*. *artemisiifolia* populations sampled in Europe and in North America. Populations are classified from a west (left) to east (right) gradient.

**Fig 4:**
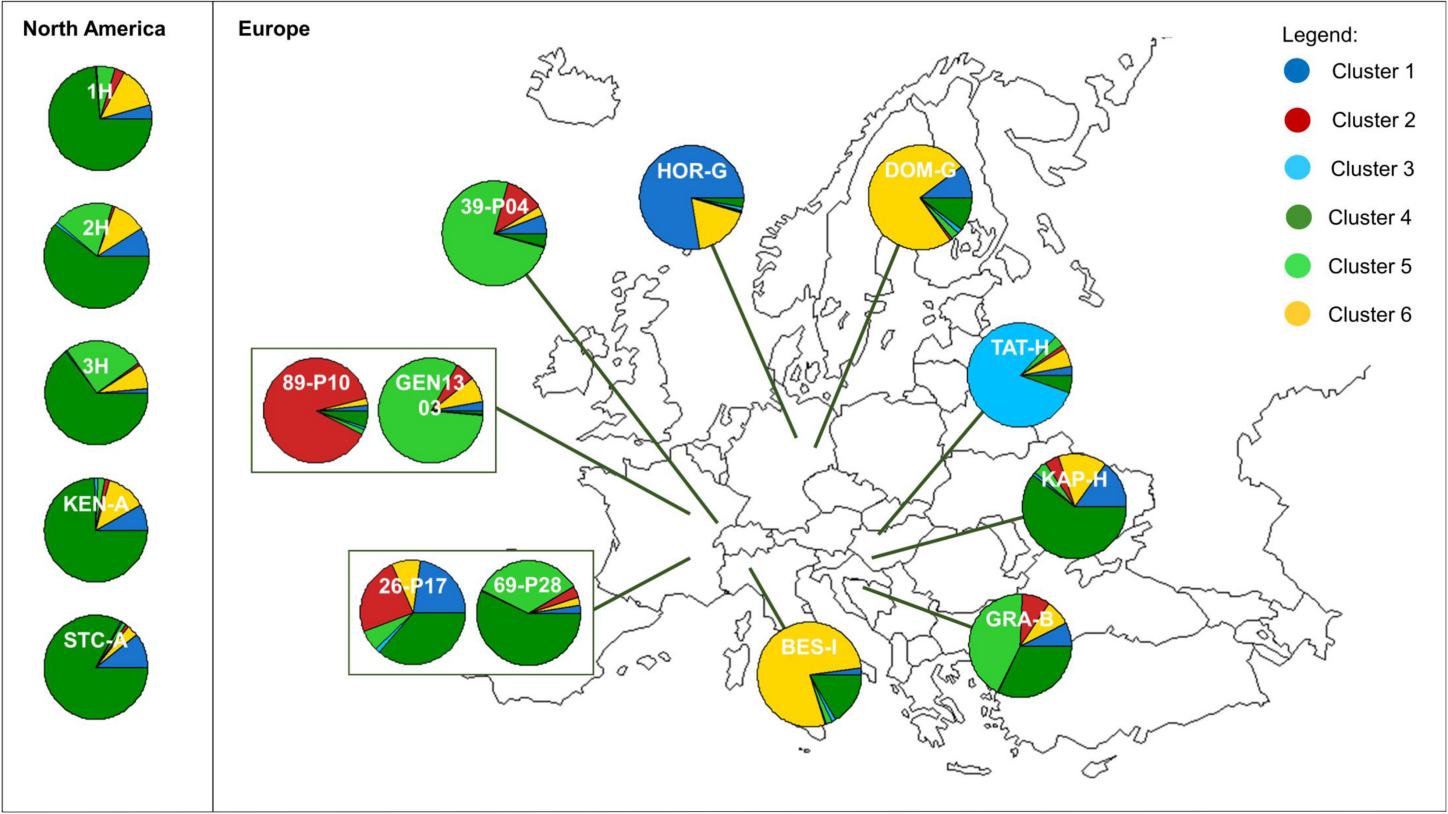
Genetic structure of 16 populations of *Ambrosia artemisiifolia* analyzed using 26 SSR markers. Proportions of the six genetic clusters within 16 *A*. *artemisiifolia* populations sampled in Europe and in North America.

The posterior likelihood generated by Structure increased continuously with *K*, whereas the Evanno method (49) suggested that the most likely number of genetic clusters was six (S3 Fig). From *K* = 2, a west-east gradient of genetic variation was observed across Europe, showing that Central and Eastern European populations are different from Western European populations, and similar to the Northern American populations studied. At *K* = 6, a more detailed genetic structuring was revealed mainly within Europe (Fig 3). Most of the additional clusters were very specific to one or two populations (cluster 1: HOR-G, cluster 2: 89-P10, cluster 3: TAT-H, cluster 6: BES-I and DOM-G, Figs 3 and 4). The two main genetic clusters (clusters 4 and 5, Fig 4) were frequent in the western and south-eastern part of the invasive range, respectively, but only the first one (cluster 4) was observed at high frequencies in the native range.

Structure analyses were also performed separately using gSSR data only or EST-SSR data only. The most likely numbers of genetic clusters among the 16 populations were four (S4 Fig) and three (S5 Fig) for EST-SSRs and gSSRs, respectively. Overall, the same patterns were observed for both datasets, i.e., variation in cluster membership probabilities among populations and a west-east gradient of genetic variation across Europe.

### Patterns of local genetic divergence and bottlenecks in the invasive range

**Fig 5:**
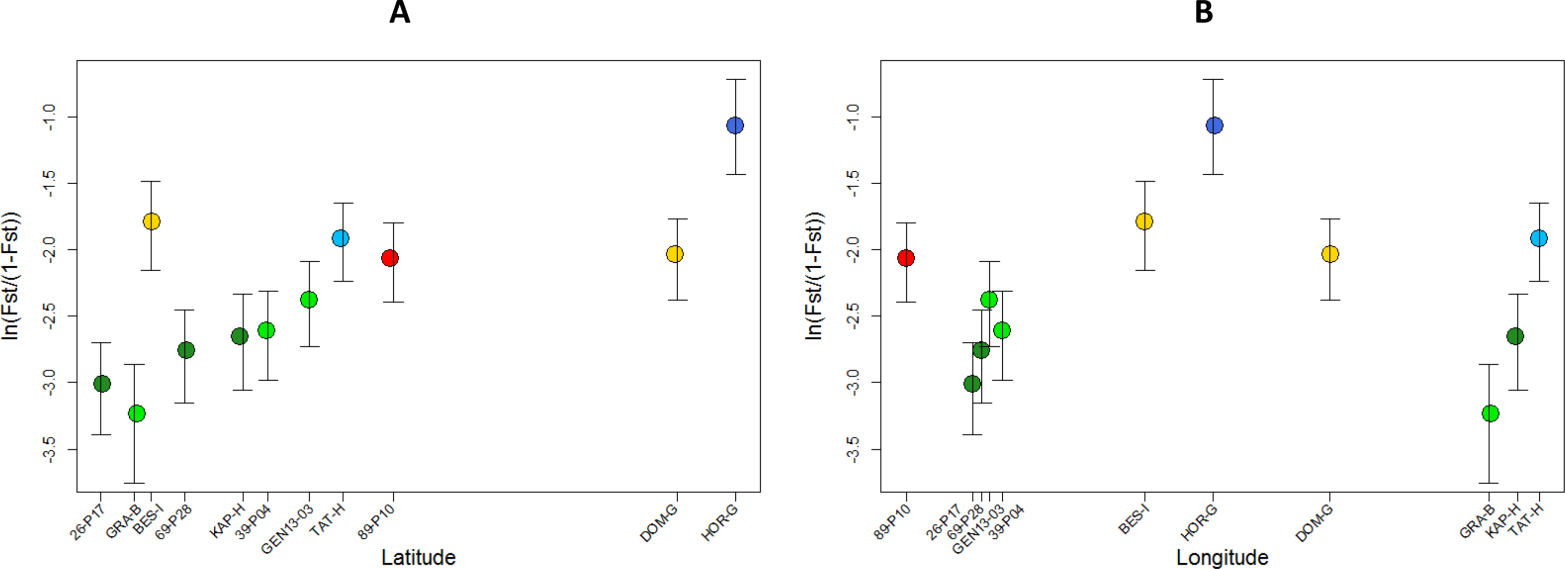
**Population-specific genetic divergence (expressed as *F***_**ST**_**/(1 –*F***_**ST**_**)) of *Ambrosia artemisiifolia* in Europe, as a function of (A) latitude and (B) longitude.** Dots are coloured according to the most frequent genetic cluster identified by Structure (at *K* = 6) in each population (see Figs 3 and 4).

Population–specific F_ST_ values were best explained by the model that included latitude as a linear explanatory factor (posterior probability: 0.66) in comparison to the null model with no explanatory factor (posterior probability: 0.28) or the model including longitude (posterior probability: 0.04). Population-specific F_ST_ values increased with latitude (Fig 5A). While there was no linear relationship with longitude, a non-linear pattern was revealed with populations from Central Europe (Italy: BES-I and Germany: HOR-G, DOM-G) and two populations located in the western (89-P10) and eastern (TAT-H) parts of the range showing elevated F_ST_ values (Fig 5B). Noticeably, these populations were those harbouring specific genetic clusters under the most detailed Structure models (Figs 3 and 4). Increased genetic divergence may be due to recent founder events for these populations. However, no significant signatures of recent bottlenecks were detected based on the Wilcoxon test for expected heterozygosity excess in any of the studied populations.

### Transferability of SSRs and relationships among species

**Fig 6:**
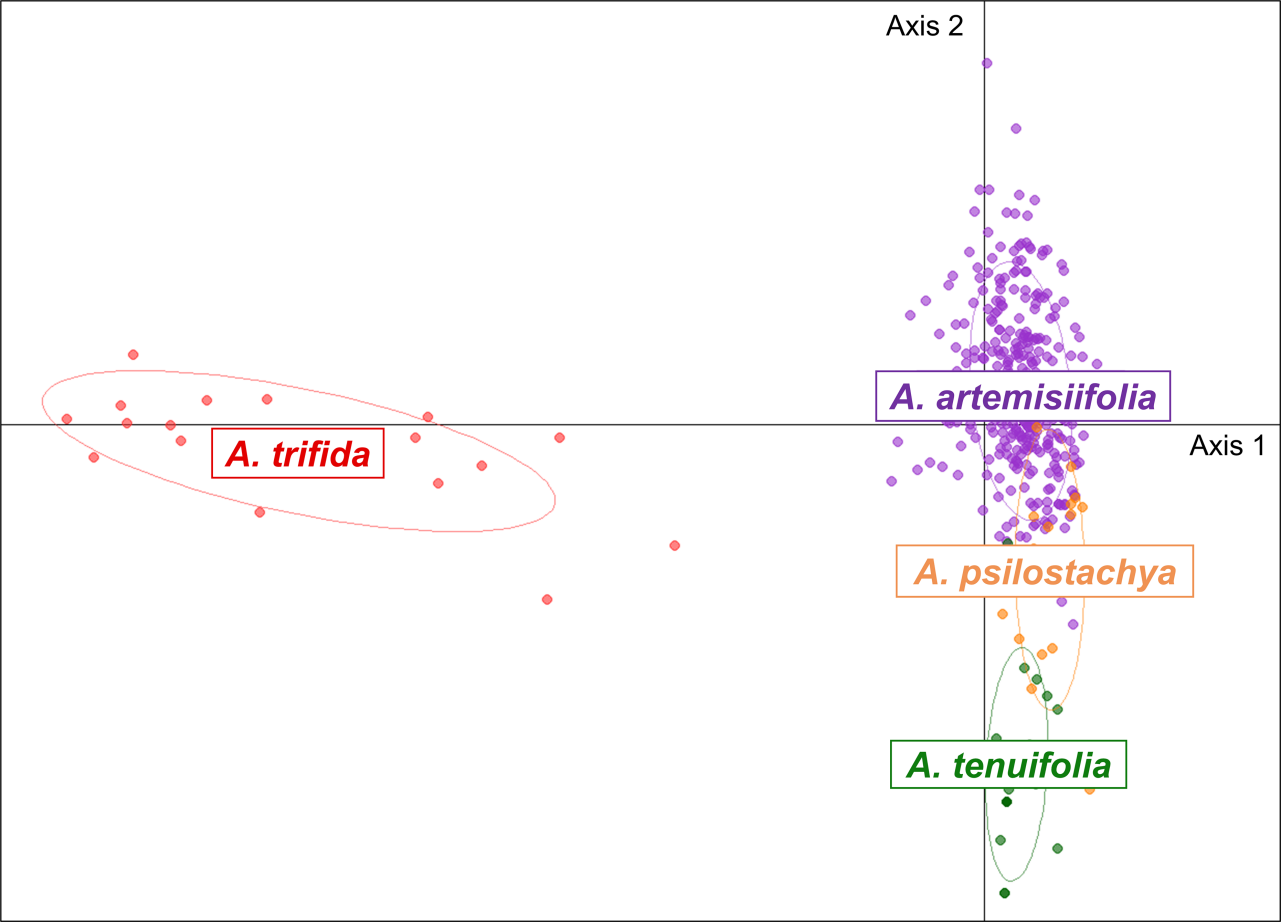
Relationships among *Ambrosia* species assessed with PCA based on eight SSR markers. Three gSSRs (SSR 17–26 and 73) and five EST-SSRs (EST-SSRs 13–61–69–111 and 123) were used. Each color represents one *Ambrosia* species.

Cross-species transferability was tested for 31 gSSRs and 32 EST-SSRs. Among these markers, 32.2%, 54.8% and 67.7% of gSSRs and 46.9%, 75% and 81.2% of EST-SSRs gave consistent amplification and clear electrophoretic migration patterns in *A*. *trifida*, *A*. *psilostachya* and *A*. *tenuifolia*, respectively (S1–S3 Tables). Among the 26 markers used to analyse the genetic variation in *A*. *artemisiifolia* (Table 3), three gSSRs (SSR17, SSR26 and SSR73) and five EST-SSRs (EST-SSR13, EST-SSR61, EST-SSR69, EST-SSR111 and EST-SSR123) were scorable in all three other species. Relationships among species were visualised by a PCA based on data at these eight markers (Fig 6). In coherence with the transferability of SSR markers, *A*. *trifida* was the most genetically divergent species, while *A*. *psilostachya* and *A*. *tenuifolia* appeared to be very genetically close to *A*. *artemisiifolia*.

## Discussion

### New, highly polymorphic nuclear SSRs in *A*. *artemisiifolia*

Next-generation sequencing technologies have considerably facilitated the development of SSRs for non-model organisms. Until recently, the method of choice was 454 sequencing of enriched gDNA libraries. 454 sequencing generates relatively long reads, which facilitates the design of primers within the regions flanking SSRs. However, the more recent Illumina sequencing technique provides much higher numbers of reads at a lower cost, and can now generate paired reads of 2×250 bp or longer [8]. Here, we implemented a rigorous initial quality filtering of reads. Further, we merged Illumina paired-end reads and kept only long-enough sequences, which facilitated primer design. A similar amplification success of potentially amplifiable loci was obtained from Illumina and from 454 data. This, together with the Illumina technology yielding one to two orders of magnitude more reads than 454, highlights Illumina as the currently most efficient sequencing technology for developing SSRs, provided reads are carefully checked for quality and paired-end reads are merged.

All markers developed were highly polymorphic in *A*. *artemisiifolia*. As compared to gSSRs, EST-SSRs are expected to be less polymorphic and more prone to the influence of selective processes: divergent selection may increase the estimation of genetic differentiation among populations at these loci, while purifying or balancing selection may have the opposite effect [9–11]. Here, allelic richness and expected heterozygosity were similar between the two kinds of markers, while genetic differentiation was slightly lower for EST-SSRs. Most EST-SSRs were tri-nucleotide repeats, for which length polymorphism does not result in any frameshift in the coding sequence. No influence of selection could be detected for any of the EST-SSRs analysed. The high level of polymorphism observed at both non-genic and genic locations in the genome of *A*. *artemisiifolia* likely reflects very large effective population sizes in a plant species known to have recently undergone a demographic expansion both in its native range [26] and in invasive ranges [15]. Similarly, a large variation for life traits is known in *A*. *artemisiifolia* [54].

### Null alleles rather than partial selfing explain excess homozygotes in *A*. *artemisiifolia*

One undesirable counterpart of high nucleotide polymorphism is the presence of null alleles resulting from mutations at primer binding sites. Here, null alleles were observed for both gSSRs and EST-SSRs, with overall estimated frequencies of about 10%. This is consistent with a literature survey indicating that null alleles frequencies are often below 20% but can in some cases range from 40% to 75% [55]. Analyzing progenies in French populations provided direct evidence for the presence of null alleles, but no evidence for selfing or biparental inbreeding. Our results were consistent with a previous study of invasive populations from China [27] that also indicated complete allogamy and no shift towards partial selfing during invasion. Significant *F*_IS_ values were observed in only seven populations out of sixteen, indicating that null alleles are the main cause for excess homozygosity. Significant *F*_IS_ values were observed in a small minority of populations from Europe but in the majority of populations from the native range. As any evolution of the mating system towards loss of selfing during invasion seems unlikely, it remains to be investigated whether this might be due to a different functioning of the populations in the two ranges, with populations from the native range showing some Wahlund effect [26].

### Genetic diversity, population structure and population-specific genetic divergence in *A*. *artemisiifolia*

Genetic diversity within population was similar in North America (the native range) and in Europe, but genetic differentiation among populations (*F*_ST_) was greater in the invasive range. A similar trend had also been observed previously [23]. This difference in *F*_ST_ values may arise simply because only a small area of the native range was sampled (five American populations) in our study and in [23]. This pattern is also consistent with a scenario involving multiple introduction events, as previously proposed [20,22,23]. Rare alleles initially present in American populations may have shifted to high frequencies in different European populations after invasion and local demographic expansion [22]. The maintenance of high levels of genetic diversity within invasive populations, a trend opposite to that found in many other biological invasions processes [56], can be attributed to high numbers of introduced seeds in multiple events [57], high gene flow and possibly genetic admixture among introduced populations [21,58].

The main pattern of population structure we observed in Europe was a west-east gradient. Differentiation between the western and eastern parts of the European invasive range had previously been observed [22] and attributed to two main, distinct invasion sources. Here, we also observed that populations from central Europe (Germany to Italy) were genetically distinct from both Western and Eastern European populations. Several genetic clusters predicted by Structure were not observed or were very infrequent in North American populations, suggesting that we may have sampled only a fraction of the native sources. Alternatively, the additional clusters revealed by Structure under the more refined model (*K* = 6) could simply reflect the elevated genetic divergence of some populations. Indeed, Structure analyses are known to be biased towards inferring extra genetic clusters when some populations have undergone strong recent genetic drift; in that case, and contrary to the assumptions of the admixture model, not all genetic clusters are ancestral sources for the present populations [59]. Population-specific genetic divergences as estimated based on the F-model largely varied among populations, a pattern not expected if all populations similarly derived from a number of ancestral sources [51]. This, together with the outputs of Structure for increasing genetic partitioning (Fig 3), suggests secondary founding events associated with genetic drift. This hypothesis was not supported by signatures for recent bottlenecks; however, it is well known that bottleneck tests have a very limited power [60]. Populations from Italy, Germany and one population from Hungary (TAT) likely had their genetic sources in the South-Eastern part of Europe, whereas one population from France (89-P10) likely originated from eastern France. Although this would need to be validated based a more extensive sampling, genetic patterns revealed here are overall consistent with two main distinct colonization events in Europe (in South-Eastern France: the Rhone valley, and South-Eastern Europe: the Pannonian plain), with secondary colonization events arising northwards and towards Central Europe.

### Genetic variation among species of the genus *Ambrosia*

Most SSR markers developed for *A*. *artemisiifolia* (65% and 75%, respectively) were transferable to *A*. *psilostachya* and *A*. *tenuifolia*, whereas only 40% were transferable to *A*. *trifida*. The genus *Ambrosia* is composed of many, not clearly delineated species [15] for which there is no well-established phylogeny. Former morphological classification considered *A*. *artemisiifolia*, *A*. *psilostachya* and *A*. *tenuifolia* as related species belonging to one same group, while *A*. *trifida* was classified in a separate group [30]. This is consistent with differences in gametophytic chromosome numbers (n = 18 for *A*. *artemisiifolia*, *A*. *psilostachya* and *A*. *tenuifolia* but n = 12 for *A*. *trifida*, [13,14,29,30,32]) and with a chloroplast DNA phylogeny [61]. The success of SSR marker transfer among species fully confirms these previous data. In addition, some degree of hybridization between *A*. *artemisiifolia* and *A*. *psilostachya* was suggested [32]. This, or homoplasy at SSR markers, may explain the overlapping genetic variation between the two species.

*A*. *psilostachya* and *A*. *tenuifolia* are perennial species that reproduce both sexually and clonally [13, 14, 32, 62]. Although these species are of less concern than the annual *A*. *artemisiifolia* and *A*. *trifida*, being less widespread and invasive, our SSR markers will be useful for assessing vegetative *versus* sexual reproduction, as well as for identifying colonization sources and relatedness among populations. However, given that several ploidy levels were reported in these species [13, 14, 29, 32], we recommend that ploidy is carefully checked before markers developed from *A*. *artemisiifolia* are transferred to *A*. *psilostachya* and *A*. *tenuifolia*. *A*. *trifida* is a noxious annual weed, very widespread in its native area [63] and introduced in several European countries including, for instance, France, Italy, Slovenia and Serbia [64,65]. Despite the potential threat set by this species on both human health and agriculture, population genetics studies are still lacking. Given its distant relation to *A*. *artemisiifolia* and the low success rate of markers transferability, we recommend that additional SSR markers are specifically developed for *A*. *trifida*.

## Conclusions

Large sets of genomic SSRs and EST-SSRs were developed and validated in *A*. *artemisiifolia*, providing useful new resources for genetic studies of this highly noxious invasive weed. All markers were highly polymorphic. EST-SSRs revealed as many alleles as gSSRs and yielded similar genetic diversity estimates. The genetic patterns revealed for a set of American and European populations confirmed results from previous studies by showing a high within-population genetic diversity in both the native and invasive ranges. A geographical gradient of genetic variation in Europe was consistent with at least two major colonization events in Western and Eastern Europe, respectively. Secondary founding events were identified, especially in Central Europe. In addition, we settled a former controversy by demonstrating that inbreeding observed within populations is attributable mostly to the presence of null alleles rather than to selfing. Last, most SSRs were transferable to three other *Ambrosia* species. These SSRs can readily be used for studying key aspects of the biology and population dynamics of the two species most closely related to *A*. *artemisiifolia* (*A*. *psilostachya* and *A*. *tenuifolia*).

## Supporting information

S1 Fig Karyotype showing the 36 chromosomes of *A*. *artemisiifolia* in a telophase (left) and in the end of a prophase (right). The red arrows indicate the chromosomes. Gx100.
(TIF)

S2 Fig **Results of Bayescan F**_**ST**_ **outlier analysis on 14 gSSR (a) and 13 EST-SSR loci (b).** The vertical bars correspond to threshold P-values of 0.05 (solid line) and 0.01 (dashed line) for the neutral model. (a) Data from all 16 populations. (b) Data from 11 European populations.
(TIF)

S3 Fig Data maximum likelihood value (left) and deltaK method (right) results used to determine the most likely number of genetic clusters with 13 EST-SSR and 13 gSSR markers. (TIF)

S4 Fig Genetic structure of 16 populations of *A*. *artemisiifolia* analyzed using the 13 EST-SSR markers. (TIF)

S5 Fig Genetic structure of 16 populations of *A*. *artemisiifolia* analyzed using the 13 gSSR markers. (TIF)

S1 Table gSSR markers obtained by 454 sequencing of enriched *A*. *artemisiifolia* gDNA and showing consistent PCR amplifications and clear electrophoretic migration patterns. Loci in bold represent the markers selected in *A*. *artemisiifolia*.
(DOCX)

S2 Table gSSR markers obtained by Illumina sequencing of raw *A*. *artemisiifolia* gDNA and showing consistent PCR amplifications and clear electrophoretic migration patterns. Loci in bold represent the markers selected in *A*. *artemisiifolia*.
(DOCX)

S3 Table EST-SSR markers obtained by 454 sequencing of *A*. *artemisiifolia* ESTs and showing consistent PCR amplifications and clear electrophoretic migration patterns. Loci in bold represent the markers selected in *A*. *artemisiifolia*.
(DOCX)

S4 Table Genetic diversity parameters at 26 nuclear SSR markers for 16 populations of *Ambrosia artemisiifolia*. *N*: number of individuals genotyped, *A*: average allelic richness after rarefaction, *H*_O_ observed heterozygosity, *H*_S_ expected heterozygosity, *F*_IS_ inbreeding coefficient estimated taking into account the presence of null alleles. * F_IS_ estimates significantly greater from zero (using the Bayesian model comparison based on Deviance Information Criterion implemented in INEST2.1).
(DOCX)
