# Supplementary figures and images for: New gSSR and EST-SSR markers reveal high genetic diversity in the invasive plant Ambrosia artemisiifolia L. and can be transferred to other invasive Ambrosia species
Source: PLoS One. 2017 May 10;12(5):e0176197. doi: 10.1371/journal.pone.0176197 (PMC5425025; doi:10.1371/journal.pone.0176197)

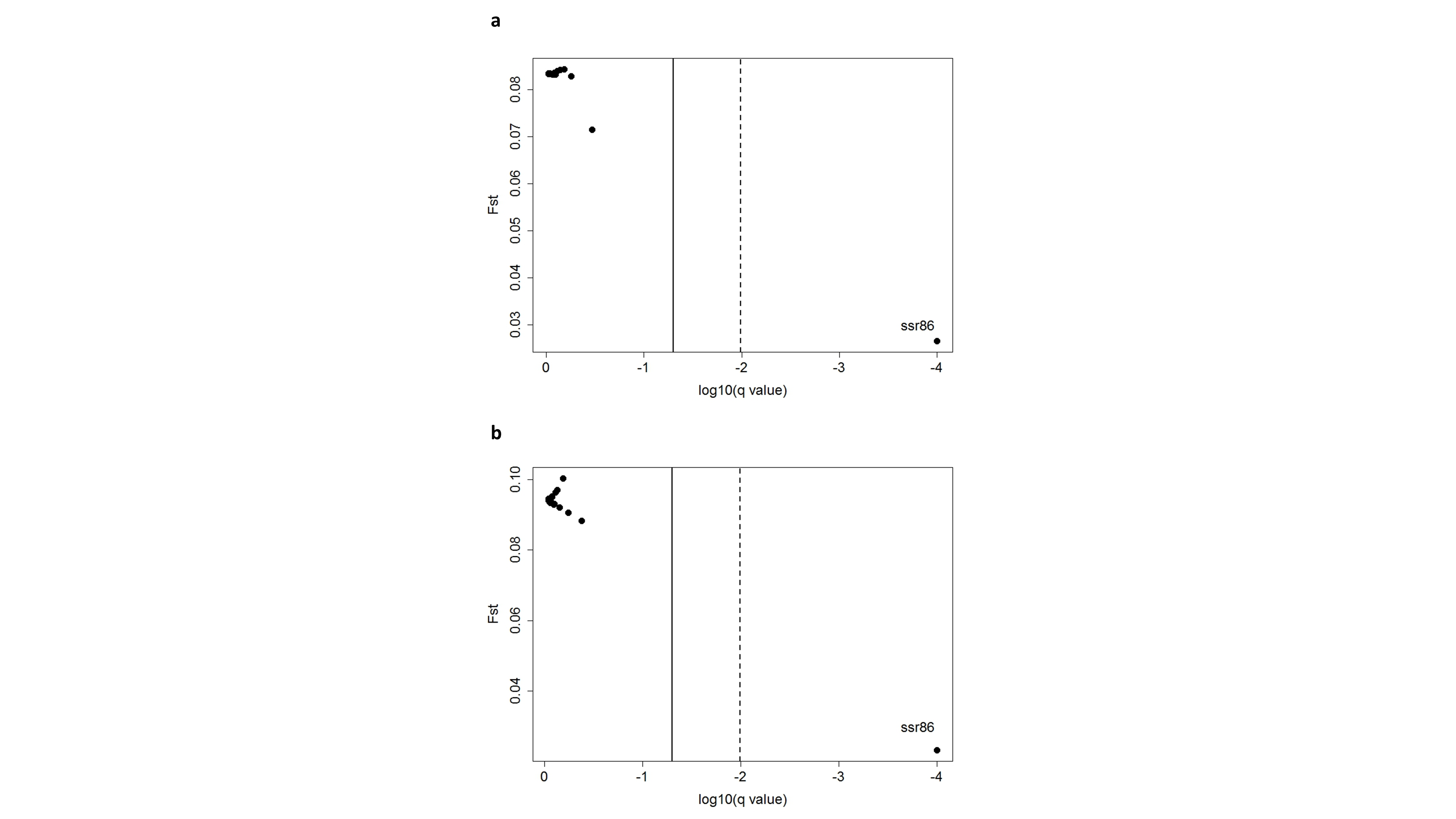

Supplement: S2 Fig — Results of Bayescan FST outlier analysis on 14 gSSR (a) and 13 EST-SSR loci (b). The vertical bars correspond to threshold P-values of 0.05 (solid line) and 0.01 (dashed line) for the neutral model. (a) Data from all 16 populations. (b) Data from 11 European populations. (TIF) [file pone.0176197.s002.tif]

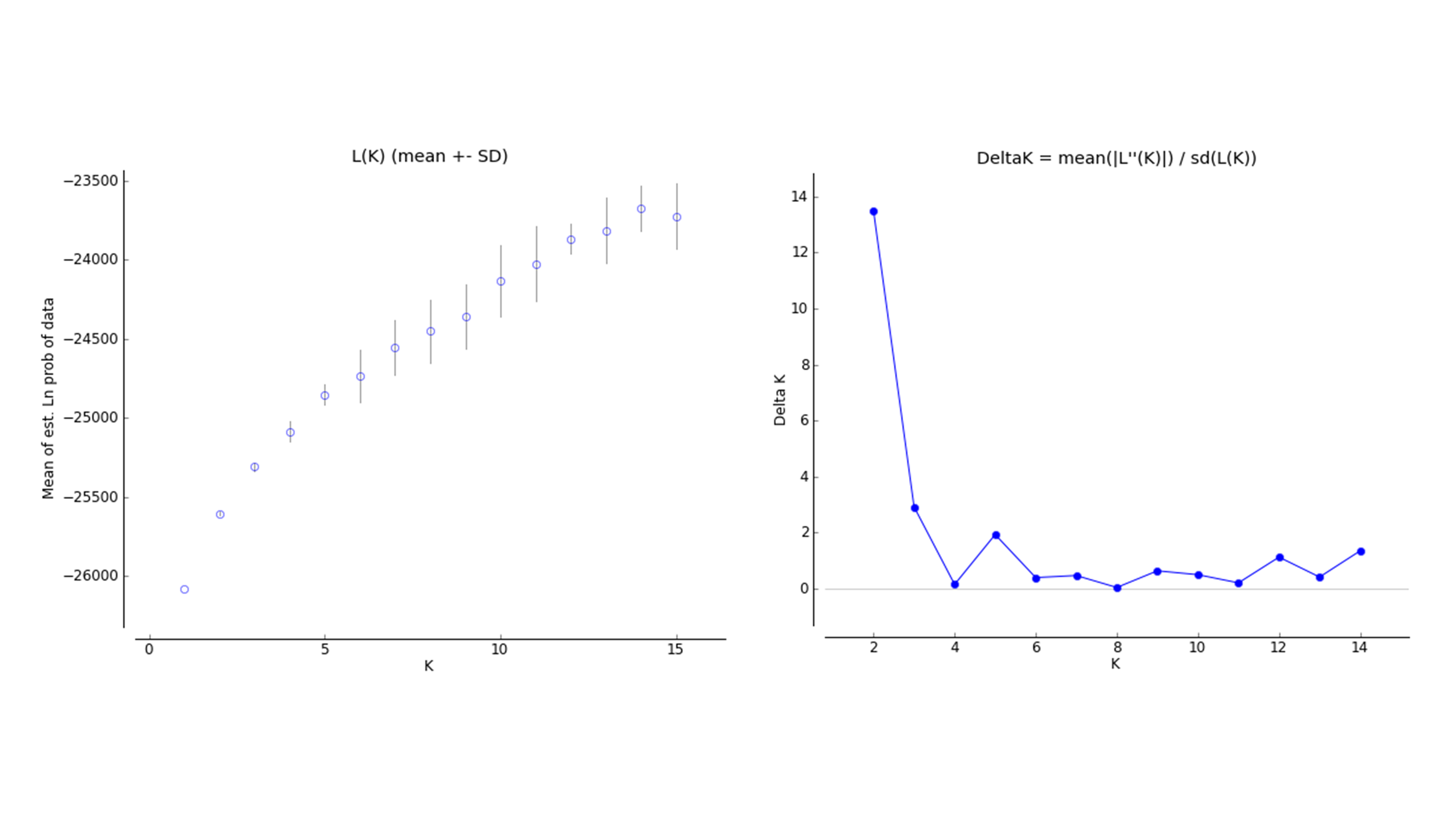

Supplement: S3 Fig — (TIF) [file pone.0176197.s003.tif]
