# Supplementary material for: New gSSR and EST-SSR markers reveal high genetic diversity in the invasive plant Ambrosia artemisiifolia L. and can be transferred to other invasive Ambrosia species
Source: PLoS One. 2017 May 10;12(5):e0176197. doi: 10.1371/journal.pone.0176197 (PMC5425025; doi:10.1371/journal.pone.0176197)
Supplement: S1 Table — Loci in bold represent the markers selected in A. artemisiifolia. (DOCX) [file pone.0176197.s006.docx]

**S1 Table. gSSR markers obtained by 454 sequencing of enriched *A. artemisiifolia* gDNA and showing consistent PCR amplifications and clear patterns.** Loci in bold represent the markers selected in *A. artemisiifolia*.

| **Locus** | **PCR product size** | **Primer left sequence** | **Primer right sequence** | **Motif length** | **Repeated motif** |
| --- | --- | --- | --- | --- | --- |
| SSR03 | 248 | GCAAACTTTTCCTGTCTGGC | AATCGATTGTTGGGTGGAAG | 5 | (CATAA)5 |
| SSR07 | 209 | AGTTATTGGATGGGGACGTG | TCCCCTCCCTAAATCCTATTTATATC | 4 | (GTTA)7 |
| SSR09 | 103 | TTAGGTATGTATTGCTTCGATTAATGT | TGCAGCTGTAAATCAGTGCAT | 4 | (TATG)7 |
| **SSR10**^a,c^ | 150 | CGTCAATGGACGATGAAGAA | CCACGTCTTCAAGAATAACAAAA | 4 | (GATA)6 |
| SSR11 | 102 | AGTGGAGGGAACTGGAAAGAA | TTGGTTGCTGACAAATCACG | 4 | (GTAT)6 |
| SSR13 | 118 | ACGGGACGTCATGATTTAGC | CAATCCCAACCTACGACCAC | 4 | (TGAA)6 |
| **SSR17**^a,b,c^ | 140 | GAACATGGATTATGAAGATGCAG | GATTAAGGTTGTCAATAAGGATTGG | 4 | (CATA)5 |
| SSR21 | 96 | GGTGATGATGGTGATTTCAATG | TTCATCATCTATCTTTCCTCCACTC | 3 | (GATA)10 |
| SSR22 | 297 | TAGCGTTCTTGTAGGGCAGC | CTGAAACAACGTCCGTGATG | 3 | (TCT)10 |
| **SSR26**^a,b,c^ | 109 | TCAAGAAATTGATTTAGAACCAAGG | GGAGAACTTGCGCTCGTATT | 3 | (GAA)9 |
| SSR28 | 243 | CCAAAACCTTGTATGTAAACCATC | GGGATGGTGGCAATTATGTC | 3 | (AAC)8 |
| SSR30 | 247 | ACCATGTCCCTAGGGTTTCC | TCTCGCTGGAACACGAATAA | 3 | (TGT)8 |
| SSR36 | 147 | AAGCCCAAAATCTTAATTGCC | TGCATAACCAAAGGTACACCA | 2 | (CA)11 |
| SSR39 | 247 | CCCGATTTCAATAGGAGTGG | GATGGCGGAGAAGGTGTAAG | 2 | (TCT)11 |
| SSR41 | 201 | AATCACAAACCACGCCAATC | AAACGATGCCTCGAACTACG | 2 | (CA)10 |
| SSR45 | 102 | TGAACAAAGAAGAGGGAAGAGG | TTCAAAACAACACAAAGAGACTCA | 2 | (GA)10 |
| **SSR47** | 96 | CAATCACCATCGTCACATCC | GGAGCCGGTCATCGTTTTAT | 2 | (AG)9 |
| SSR54 | 183 | GGCAAGGGTTTTGGGTTAGT | GAGAGCGTCATTGCTACGTG | 2 | (CT)9 |
| SSR61 | 190 | TTCCAACACCCAAACCCTAC | GGTCAGTTTCCATCACCGTC | 2 | (TC)9 |
| SSR64 | 305 | CCAAGATTTATGATCGCGAAA | TGTCATATACGGACGGAGGT | 2 | (TG)9 |
| SSR66 | 113 | GGCAGTCGGATATCTGCTTT | GAGCGTGATTTCTTCAACGA | 3 | (CTT)7 |
| **SSR67**^b,c^ | 201 | ACAAAGCCACTTTTGATGCC | CCTTCAGATGTTTGGCCTTC | 3 | (GAA)7 |
| SSR68 | 123 | TGCTTGGTAAACTCTATAATGAAGCTC | TCTCATCATTTCCTTCTTCATCAG | 3 | (GAA)7 |
| SSR70 | 151 | AAGGTTTGCCTACCTCCCAC | AGTCAATTCAACCTGACCCG | 3 | (GTT)7 |
| **SSR71**^b^ | 132 | GACTTTCGCTTCCCAAACAC | CAAATGTCATGGGGAGAAGG | 3 | (TCC)7 |
| **SSR73**^a,b,c^ | 180 | GACTCATGCATATGGAACACG | CCAAATGGTCTACCTCCTGC | 2 | (AC)8 |
| SSR74 | 132 | CACCCATACAACATCACACCC | CGGTGTTAGAGAACCGTCTTC | 2 | (AC)8 |
| SSR85 | 213 | ACGGCGTTGGATGATTTTAG | TTCGGTGTTATGATTCGCAG | 2 | (GA)8 |
| **SSR86** | 190 | TCTGCCTTCTTTGAGGATCTTT | AAAATACCTGCCTATCATGGTTGA | 2 | (GA)8 |
| **SSR91** | 98 | AAACATCTTTCGATTCAAGCTCA | TGGTTTGGATATTGATAGAACAGC | 2 | (TC)8 |

^a^Marker also amplified (clear patterns) in *A. trifida*; ^b^Marker also amplified (clear patterns) in *A. psilostachya*; ^c^Marker also amplified (clear patterns) in *A. tenuifolia*
