# Supplementary material for: New gSSR and EST-SSR markers reveal high genetic diversity in the invasive plant Ambrosia artemisiifolia L. and can be transferred to other invasive Ambrosia species
Source: PLoS One. 2017 May 10;12(5):e0176197. doi: 10.1371/journal.pone.0176197 (PMC5425025; doi:10.1371/journal.pone.0176197)
Supplement: S2 Table — Loci in bold represent the markers selected in A. artemisiifolia. (DOCX) [file pone.0176197.s007.docx]

**S2 Table. gSSR markers obtained by Illumina sequencing of raw *A. artemisiifolia* gDNA and showing consistent PCR amplifications and clear patterns.** Loci in bold represent the markers selected in *A. artemisiifolia*.

| **Locus** | **PCR product size** | **Primer left sequence** | **Primer right sequence** | **Motif lenght** | **Repeated motif** |
| --- | --- | --- | --- | --- | --- |
| **ILL02**^b^ | 274 | AGCTGCCTTTGCATACCTTG | AGCGGTTGGTGGAATACTTG | 6 | (ACCACT)6 |
| ILL09^c^ | 107 | TGCAATGCATACCTTGGAAC | CTGCCCATGTTTACCGATTC | 5 | (AATAC)6 |
| **ILL12**^a,b^ | 132 | CCCATTCCCGACAATAACAG | TGATGAAAGAAAGGTATAAGCAGC | 5 | (AACAG)5 |
| ILL18^a,b,c^ | 150 | CCGTTTCACAGGGTTTGTTC | ATGGTCCCAGTTCAATCCAG | 4 | (AAAT)5 |
| ILL29^b,c^ | 144 | AACCGCTATAGCACATTAGCAAC | GGCTCTGGAAAGACCCTTG | 4 | (AAAC)5 |
| ILL34 | 171 | GGAATACCTTGATGAACGCC | AACATTCAAGGCTACAATGCC | 4 | (AAAT)5 |
| ILL35^b,c^ | 182 | CAGGCATGGTGAGTAGGAGC | CTTATAACACCCGGCAAGG | 4 | (AATG)5 |
| ILL37^c^ | 137 | ACCGGAGCTATGGCAGTAAG | AACGTCCATACAGGGTCTCC | 3 | (AAT)17 |
| ILL39 | 288 | CGCCTGACTAGCCACCTCTAC | ACTCTGTTGGGAACTGGCTC | 3 | (AAT)14 |
| ILL40^a,b,c^ | 177 | GAGATCGGAACAGAGCAAGG | GTCAAACAGCATCACTCGCC | 3 | (AAT)13 |
| ILL41^c^ | 150 | TAACCTTAGCCAATCAAATAAGGG | TTCATTATTGATGCCCGTTG | 3 | (AAG)13 |
| **ILL48**^c^ | 121 | GCACGCCATTCTTCACTTATG | GAGGGCGAAATAGGGTAATTG | 3 | (AGC)10 |
| ILL56^b,c^ | 132 | CGGATATCGAAACAATGCAG | GGCGCTTCATGTACACCTAAC | 3 | (AAT)8 |
| ILL57^a,b,c^ | 257 | AAGCTTCCCTGCAGTCCTTAC | TGGCATAGCAGTTTAAAGATTGTG | 3 | (AAT)8 |
| **ILL64**^b,c^ | 272 | CCCTGTTTCCAAAGACATCC | CATTAGTAGCCGCCTCAAGC | 3 | (ACC)7 |
| ILL71^a,b,c^ | 252 | GATTCAACCGTAGGGAAGCC | CATATTCCGGCCCATTTAAC | 3 | (AAT)7 |
| ILL73^a,c^ | 162 | TCTTCTTTGCCGTTACCGAG | TGAAATGTCCCTCCATCAAC | 3 | (ACC)7 |
| **ILL74**^b,c^ | 272 | AGCCTGGCCTTCAGGTATG | GTCCACTTCTGCCACCTCTG | 3 | (AGC)7 |
| ILL75^b,c^ | 160 | CGAGCCTTCAATACCTATTTGG | GGCTACCCAATAACTTCCACC | 3 | (AAT)7 |
| ILL79 | 250 | TGTCAAACCAATTTAGTCAAGGC | TTGGAAAGAGGATCAGTGAAGG | 3 | (AAT)7 |
| ILL84 | 248 | TGTAATTTGAAGGTATTGGCCC | GAGGAGCTTTGCATAGGGTG | 2 | (AG)15 |
| ILL95^c^ | 147 | CTACCAGCACGGTAACATCG | TTTGATTTGAGGAAATTTGGG | 2 | (AG)12 |

^a^Marker also amplified (clear patterns) in *A. trifida*; ^b^Marker also amplified (clear patterns) in *A. psilostachya*; ^c^Marker also amplified (clear patterns) in *A. tenuifolia*
