# Supplementary material for: New gSSR and EST-SSR markers reveal high genetic diversity in the invasive plant Ambrosia artemisiifolia L. and can be transferred to other invasive Ambrosia species
Source: PLoS One. 2017 May 10;12(5):e0176197. doi: 10.1371/journal.pone.0176197 (PMC5425025; doi:10.1371/journal.pone.0176197)
Supplement: S3 Table — Loci in bold represent the markers selected in A. artemisiifolia. (DOCX) [file pone.0176197.s008.docx]

**S3 Table. EST-SSR markers obtained by 454 sequencing of *A. artemisiifolia* ESTs and showing consistent PCR amplifications and clear patterns.** Loci in bold represent the markers selected in *A. artemisiifolia*.

| **Locus** | **PCR product size** | **Primer left sequence** | **Primer right sequence** | **Motif length** | **Repeated motif** | **Gene homology (discontiguous megablast)** | **E-value^d^** |
| --- | --- | --- | --- | --- | --- | --- | --- |
| EST11^b^ | 228 | ACCCCACTGTAAGCTCAAGG | GCCTTCTGCAGACCATCTTC | 3 | (AGT)10 |  |  |
| **EST13**^a,b,c^ | 177 | CCCAACCACCATAAAACACC | GTTACCGGCAGCTGTAGGAG | 3 | (AGT)9 |  |  |
| EST21^a,b,c^ | 223 | GAGAAAGCGGTTACAGCGTC | CAACCGGTAACCATACCCAC | 3 | (ACC)8 | *Lactuca sativa* 4-hydroxyphenylpyruvate dioxygenase mRNA | 7e-128 |
| EST40^c^ | 105 | TCCTCTACACTCCCAAACGG | ACGGTTGTTGATCGGAGAAG | 2 | (AG)9 | *Populus trichocarpa* actin-depolymerizing factor family protein | 2e-29 |
| **EST50**^b^ | 193 | TACAGTTCCAGTTCCCCTGC | AAAGAGCCTTCACCTCATCG | 4 | (AAAG)5 |  |  |
| EST54^b,c^ | 314 | GAGAATTGCGTTTGCGAAG | TTGTTGGTCAACGGAAGATG | 4 | (AATC)5 | *Populus trichocarpa* epidermal differentiation family protein | 2e-25 |
| **EST56**^a,c^ | 200 | GCTGCACATTCTTCCTCTCC | ATCAAATTTCCCCTTCCACC | 3 | (AGC)7 |  |  |
| **EST61**^a,b,c^ | 145 | CCACGTCATCATTCCTACCC | GATGAGGCGGTGAAGAAGAG | 3 | (AAT)7 | *Theobroma cacao* Nucleobase-ascorbate transporter | 6e-18 |
| EST64 | 146 | CGCGATTCAACCTCTTCTTC | GCCAAAGCTTGTTCCAAATC | 3 | (ACT)7 |  |  |
| EST65^a,c^ | 123 | CCCATCAATCCAAAACCTTG | GGGATGAGAGCTGTGGAGAC | 3 | (AGT)7 |  |  |
| **EST69**^a,b,c^ | 108 | TGTGAAAACGAGGGTTAGGG | AAGCAACGCAAACCAAAGAG | 3 | (AAT)7 | *Solanum lycopersicum* rho GTPase-activating protein 2-like | 3e-31 |
| **EST71**^b,c^ | 123 | CCAACACCTTCAAACCCATC | CCGGCAATAACCACTAGCTC | 3 | (AAT)7 | *Medicago truncatula* twin arginine translocase | 8e-39 |
| EST74^a,b,c^ | 172 | TCGATGACAATCTCGCAGAC | AACGGTTCAGACACTCCACC | 3 | (ACT)6 |  |  |
| EST85^b,c^ | 189 | GACGAACCTCAACCTTCAGC | TTTGTTGGCTTTGGATCCTC | 3 | (AAT)6 | *Theobroma cacao* Tetratricopeptide repeat-like superfamily protein | 3e-17 |
| EST89^a,b,c^ | 190 | AGCATTGGTGAGTTTGGTCC | TTTAGGTCGATCGAGTTGCC | 3 | (AAG)6 | *Populus trichocarpa* transcription initiation factor-related family protein | 3e-37 |

^a^Marker also amplified (clear patterns) in *A. trifida*; ^b^Marker also amplified (clear patterns) in *A. psilostachya*; ^c^Marker also amplified (clear patterns) in *A. tenuifolia;* ^d^E-value: E-value<10e-10 means that there is a potential homology

| **Locus** | **PCR product size** | **Primer left sequence** | **Primer right sequence** | **Motif length** | **Repeated motif** | **Gene homology (discontiguous megablast)** | **E-value^a^** |
| --- | --- | --- | --- | --- | --- | --- | --- |
| **EST111**^a,b,c^ | 123 | AGAAACACTCCGGCTCACTG | CATTTGGTCAAACACGGATG | 3 | (ACC)7 | *Castanea sativa* endochitinase | 5e-14 |
| **EST113**^b,c^ | 142 | TTCTTCGTGAGTCCATGGTG | ATCACCGTCATTACCCCAAG | 3 | (ACC)7 | *Solanum lycopersicum* trihelix transcription factor ASIL1 | 1e-30 |
| EST114 ^b,c^ | 108 | TTTCGGTCACCAGTGTCAAC | ACACCCAAATCAGCATACCC | 3 | (AGT)7 |  |  |
| EST121^b,c^ | 172 | TCATCTAATCCATCCGCCTC | TCGGTTTCACGAGATCAGC | 3 | (AAT)7 | *Malus x domestica* chloride channel protein CLC-d-like | 4e-16 |
| **EST123**^a,b,c^ | 112 | CTTCATGATTGAACCATCGG | CAACAATGGCCACAACTCAC | 3 | (ACC)7 | *Malus x domestica* MYBR domain class transcription factor | 5e-22 |
| EST126^b^ | 119 | AAAGAGGCCGCAGATGTATG | GCGTCTATGGAAAGAGAGGG | 3 | (ACC)7 |  |  |
| **EST131^c^** | 129 | ATCGGTTTTCGGTGTTTCAC | CCCAGGAAGTATTCTCCGTC | 3 | (ACC)7 | *Erythranthe guttatus* AT-hook motif nuclear-localized protein 8-like | 2e-22 |
| **EST135**^b,c^ | 122 | AATCGTGTTGCTTTGATGCC | AATCAAGCGCCGATTCTTAC | 3 | (ACG)7 |  |  |
| **EST138**^a^ | 128 | TTATCCCGTTACCGACAACC | CGTCGTTGTAGAACACGACAC | 3 | (ACC)7 | *Solanum lycopersicum* serine/threonine protein kinase pk23 | 2e-16 |
| EST141^b,c^ | 158 | TTGGCCAATGATCGCTTTAG | AGCGGAATGGCAGGTAAAC | 3 | (AAT)7 |  |  |
| EST149^b,c^ | 145 | GAACGCTGTCTTCTTGGAGG | GGTGACCGTGCTTTTAGAGG | 3 | (AAG)6 | *Populus trichocarpa* U5 small nuclear ribonucleoprotein helicase | 3e-122 |
| EST150^a,b,c^ | 128 | ATAGCGACGATGAAGCCAAC | TGGACCGATATCAAAGGCTC | 3 | (AAT)6 | *Ricinus communis* zinc finger protein | 1e-47 |
| EST151^a^ | 123 | AATCGCAATCAACACTGTGC | TCCTCAGGATGATCGGAAAC | 3 | (AAT)6 |  |  |
| **EST153**^a,c^ | 164 | CACCAATAATCCCAACCGAC | GGATGATGGAGCAAGAGGAG | 3 | (AGG)6 | *Morus notabilis* GTP-binding protein ERG | 3e-95 |
| EST154^b,c^ | 186 | TGGGTAACCGCTTAAGTTGC | ACCCATCAAACCGTTGAGAC | 3 | (AAT)6 | *Nicotiana sylvestris* putative methylesterase 14 | 2e-18 |
| EST167^a,b,c^ | 142 | CACCTTCAGCCTTAACCACC | CGCTTTCCTTCACCTCAGTC | 3 | (ACG)6 | *Populus trichocarpa* SWIB complex BAF60b domain-containing family protein | 3e-32 |
| EST173^b,c^ | 105 | AAGAAAGGGAACGGAAAAGC | GAATGCAATCCCACCAAAAC | 3 | (CCG)6 |  |  |

^a^Marker also amplified (clear patterns) in *A. trifida*; ^b^Marker also amplified (clear patterns) in *A. psilostachya*; ^c^Marker also amplified (clear patterns) in *A. tenuifolia;* ^d^E-value: E-value<10e-10 means that there is a potential homology
