# Supplementary material for: New gSSR and EST-SSR markers reveal high genetic diversity in the invasive plant Ambrosia artemisiifolia L. and can be transferred to other invasive Ambrosia species
Source: PLoS One. 2017 May 10;12(5):e0176197. doi: 10.1371/journal.pone.0176197 (PMC5425025; doi:10.1371/journal.pone.0176197)
Supplement: S4 Table — N: number of individuals genotyped, A: average allelic richness after rarefaction, HO observed heterozygosity, HS expected heterozygosity, FIS inbreeding coefficient estimated taking into account the presence of null alleles. * FIS estimates significantly greater from zero (using the Bayesian model comparison based on Deviance Information Criterion implemented in INEST2.1). (DOCX) [file pone.0176197.s009.docx]

**S4 Table. Genetic diversity parameters at 26 nuclear SSR markers for 16 populations of *Ambrosia artemisiifolia*.** *N*: number of individuals genotyped, *A*: average allelic richness after rarefaction, *H*_O_ observed heterozygosity, *H*_S_ expected heterozygosity, *F*_IS_ inbreeding coefficient estimated taking into account the presence of null alleles. * *F*_IS_ estimates significantly greater from zero (using the Bayesian model comparison based on Deviance Information Criterion implemented in INEST2.1).

| Population | *N* | *A* | *H*_O_ | *H*_S_ | *F_I_*_S_ |
| --- | --- | --- | --- | --- | --- |
| 1H | 16 | 4.212 | 0.488 | 0.665 | 0.000 |
| 2H | 11 | 4.260 | 0.578 | 0.657 | 0.110* |
| 3H | 15 | 3.934 | 0.505 | 0.611 | 0.077* |
| KEN | 24 | 4.462 | 0.463 | 0.664 | 0.104* |
| STC | 20 | 4.096 | 0.518 | 0.657 | 0.168* |
| 26P17 | 24 | 4.412 | 0.523 | 0.670 | 0.234* |
| 39P04 | 24 | 4.030 | 0.522 | 0.656 | 0.000 |
| 69P28 | 24 | 4.095 | 0.497 | 0.647 | 0.000 |
| 89P10 | 24 | 3.898 | 0.492 | 0.618 | 0.094 |
| GEN13.03 | 24 | 3.988 | 0.468 | 0.629 | 0.081* |
| BES | 20 | 3.404 | 0.434 | 0.554 | 0.075 |
| HOR | 24 | 3.027 | 0.404 | 0.522 | 0.000 |
| DOM | 24 | 3.751 | 0.465 | 0.602 | 0.052 |
| TAT | 20 | 3.644 | 0.560 | 0.617 | 0.000 |
| KAP | 20 | 4.204 | 0.529 | 0.644 | 0.182* |
| GRA | 20 | 4.400 | 0.564 | 0.665 | 0.000 |
